# Omicron-Associated Changes in Severe Acute Respiratory Syndrome Coronavirus 2 (SARS-CoV-2) Symptoms in the United Kingdom

**DOI:** 10.1093/cid/ciac613

**Published:** 2022-08-19

**Authors:** Karina-Doris Vihta, Koen B Pouwels, Tim E A Peto, Emma Pritchard, Thomas House, Ruth Studley, Emma Rourke, Duncan Cook, Ian Diamond, Derrick Crook, David A Clifton, Philippa C Matthews, Nicole Stoesser, David W Eyre, Ann Sarah Walker, COVID-19 Infection Survey team, I D, E R, Ruth Studley, Tina Thomas, D Cook, Daniel Ayoubkhani, Russell Black, Antonio Felton, Megan Crees, Joel Jones, Lina Lloyd, Esther Sutherland, A S W, D Crook, P C M, T E A P, E P, N S, K D V, Jia Wei, Alison Howarth, George Doherty, James Kavanagh, Kevin K Chau, Hatch Stephanie B, Daniel Ebner, Lucas Martins Ferreira, Thomas Christott, Brian D Marsden, Wanwisa Dejnirattisai, Juthathip Mongkolsapaya, Sarah Cameron, Phoebe Tamblin-Hopper, Magda Wolna, Rachael Brown, Sarah Hoosdally, Richard Cornall, Yvonne Jones, David I Stuart, Gavin Screaton, Katrina Lythgoe, David Bonsall, Tanya Golubchik, Helen Fryer, John Bell, Kevin Paddon, Tim James, John Newton, Julie Robotham, Paul Birrell, Helena Jordan, Tim Sheppard, Graham Athey, Dan Moody, Leigh Curry, Pamela Brereton, Ian Jarvis, Anna Godsmark, George Morris, Bobby Mallick, Phil Eeles, Jodie Hay, Jessica Lee, White Sean, Tim Evans, Lisa Bloemberg, Katie Allison, Anouska Pandya, Sophie Davis, David I Conway, Margaret MacLeod, Chris Cunningham

**Affiliations:** Nuffield Department of Medicine, University of Oxford, Oxford, United Kingdom; The National Institute for Health Research Health Protection Research Unit in Healthcare Associated Infections and Antimicrobial Resistance at the University of Oxford, Oxford, United Kingdom; Department of Engineering, University of Oxford, Oxford, United Kingdom; The National Institute for Health Research Health Protection Research Unit in Healthcare Associated Infections and Antimicrobial Resistance at the University of Oxford, Oxford, United Kingdom; Health Economics Research Centre, Nuffield Department of Population Health, University of Oxford, Oxford, United Kingdom; Nuffield Department of Medicine, University of Oxford, Oxford, United Kingdom; The National Institute for Health Research Health Protection Research Unit in Healthcare Associated Infections and Antimicrobial Resistance at the University of Oxford, Oxford, United Kingdom; The National Institute for Health Research Oxford Biomedical Research Centre, University of Oxford, Oxford, United Kingdom; Department of Infectious Diseases and Microbiology, Oxford University Hospitals NHS Foundation Trust, John Radcliffe Hospital, Oxford, United Kingdom; Nuffield Department of Medicine, University of Oxford, Oxford, United Kingdom; The National Institute for Health Research Health Protection Research Unit in Healthcare Associated Infections and Antimicrobial Resistance at the University of Oxford, Oxford, United Kingdom; Department of Mathematics, University of Manchester, Manchester, United Kingdom; IBM Research, Hartree Centre, Sci-Tech Daresbury, Daresbury, United Kingdom; Office for National Statistics, Newport, United Kingdom; Office for National Statistics, Newport, United Kingdom; Office for National Statistics, Newport, United Kingdom; Office for National Statistics, Newport, United Kingdom; Nuffield Department of Medicine, University of Oxford, Oxford, United Kingdom; The National Institute for Health Research Health Protection Research Unit in Healthcare Associated Infections and Antimicrobial Resistance at the University of Oxford, Oxford, United Kingdom; The National Institute for Health Research Oxford Biomedical Research Centre, University of Oxford, Oxford, United Kingdom; Department of Infectious Diseases and Microbiology, Oxford University Hospitals NHS Foundation Trust, John Radcliffe Hospital, Oxford, United Kingdom; Department of Engineering, University of Oxford, Oxford, United Kingdom; Nuffield Department of Medicine, University of Oxford, Oxford, United Kingdom; Francis Crick Institute, London, United Kingdom; Division of Infection and Immunity, University College London, London, United Kingdom; Department of Infection, University College London Hospitals, London, United Kingdom; Nuffield Department of Medicine, University of Oxford, Oxford, United Kingdom; The National Institute for Health Research Health Protection Research Unit in Healthcare Associated Infections and Antimicrobial Resistance at the University of Oxford, Oxford, United Kingdom; The National Institute for Health Research Oxford Biomedical Research Centre, University of Oxford, Oxford, United Kingdom; Department of Infectious Diseases and Microbiology, Oxford University Hospitals NHS Foundation Trust, John Radcliffe Hospital, Oxford, United Kingdom; The National Institute for Health Research Health Protection Research Unit in Healthcare Associated Infections and Antimicrobial Resistance at the University of Oxford, Oxford, United Kingdom; The National Institute for Health Research Oxford Biomedical Research Centre, University of Oxford, Oxford, United Kingdom; Big Data Institute, Nuffield Department of Population Health, University of Oxford, Oxford, United Kingdom; Nuffield Department of Medicine, University of Oxford, Oxford, United Kingdom; The National Institute for Health Research Health Protection Research Unit in Healthcare Associated Infections and Antimicrobial Resistance at the University of Oxford, Oxford, United Kingdom; The National Institute for Health Research Oxford Biomedical Research Centre, University of Oxford, Oxford, United Kingdom

**Keywords:** SARS-CoV-2, Omicron, symptoms

## Abstract

**Background:**

The severe acute respiratory syndrome coronavirus 2 (SARS-CoV-2) Delta variant has been replaced by the highly transmissible Omicron BA.1 variant, and subsequently by Omicron BA.2. It is important to understand how these changes in dominant variants affect reported symptoms, while also accounting for symptoms arising from other cocirculating respiratory viruses.

**Methods:**

In a nationally representative UK community study, the COVID-19 Infection Survey, we investigated symptoms in polymerase chain reaction (PCR)–positive infection episodes versus PCR-negative study visits over calendar time, by age and vaccination status, comparing periods when the Delta, Omicron BA.1, and BA.2 variants were dominant.

**Results:**

Between October 2020 and April 2022, a total of 120 995 SARS-CoV-2 PCR-positive episodes occurred in 115 886 participants, with 70 683 (58%) reporting symptoms. The comparator comprised 4 766 366 PCR-negative study visits (483 894 participants), with symptoms reported at 203 422 visits (4%). Symptom reporting in PCR-positive infections varied over time, with a marked reduction in loss of taste/smell as Omicron BA.1 dominated, which was maintained with BA.2 (44% symptomatic infections reporting loss of taste/45% symptomatic infections reporting loss of smell on 17 October 2021, 16%/13% 2 January 2022, 15%/12% 27 March 2022). Cough, fever, shortness of breath, myalgia, fatigue/weakness, and headache also decreased after Omicron BA.1 dominated, but sore throat increased, the latter to a greater degree than concurrent increases in PCR-negative visits. Fatigue/weakness increased again after BA.2 dominated, although to a similar degree to concurrent increases in PCR-negative visits. Symptoms were consistently more common in adults aged 18–65 years than in children or older adults.

**Conclusions:**

Increases in sore throat (also common in the general community), along with a marked reduction in loss of taste/smell, make Omicron harder to detect with symptom-based testing algorithms, with implications for institutional and national testing policies.

## Teaser

In a UK community study, loss of taste/smell was markedly less commonly reported with Omicron BA.1/BA.2 than with Delta severe acute respiratory syndrome coronavirus 2 infections, with smaller declines in reported shortness of breath, myalgia, and fatigue/weakness but increases in sore throat, challenging symptom-based testing algorithms.

Highly-transmissible severe acute respiratory syndrome coronavirus 2 (SARS-CoV-2) Omicron variants, BA.1 and BA.2, emerged and become dominant at the end and start of 2021 and 2022, coincident with other winter respiratory viruses circulating in the Northern hemisphere, changes in symptomatology may influence clinical and testing policy. Experimental and clinical data suggest that Omicron has less impact on the lower respiratory tract, leading to less severe disease [1–7], with the variant-defining mutations potentially also affecting other symptoms.

We used the UK COVID-19 Infection Survey, a nationally representative longitudinal household study [8], to investigate if SARS-CoV-2 infection symptoms have changed with the Omicron variants. We compared the probability of reporting any symptoms, as well as the probability of reporting specific symptoms in both SARS-CoV-2 polymerase chain reaction (PCR)–positive infection episodes and comparator PCR-negative study visits, focusing on time periods when the Delta variant (described previously only to August 2021 [9]), Omicron BA.1, and Omicron BA.2 were dominant in the United Kingdom [10].

## METHODS

This analysis was based on SARS-CoV-2 PCR tests of nose and throat swab samples taken regularly between 1 October 2020 and 23 April 2022 from participants in the Office for National Statistics COVID-19 Infection Survey (ISRCTN21086382; https://www.ndm.ox.ac.uk/COVID-19/COVID-19-infection-survey/protocol-and-information-sheets). The survey has invited private households to enroll on a continuous basis, selected at random from address lists and previous surveys to provide a representative UK sample, described in detail elsewhere [8, appendix]. Participant characteristics and representativeness are also presented in detail elsewhere [9, appendix], illustrating that the sample broadly represents the wider population. After receipt of verbal agreement to participate, a study worker visited each household to obtain written informed consent, from parents/carers for those aged 2–15 years; children aged 10–15 years also provided written assent. Children <2 years old were not eligible, to avoid asking parents to swab infants and very young children. Ethical approval was provided by the South Central Berkshire B Research Ethics Committee (no. 20/SC/0195).

Individuals were asked about demographics, symptoms, contacts and relevant behaviors (https://www.ndm.ox.ac.uk/COVID-19/COVID-19-infection-survey/case-record-forms). Participants ≥12 years old self-collected nose and throat swab samples, following study worker instructions, to reduce transmission risks. Parents/carers obtained swab samples from children 2–11 years old. At the first visit, participants were asked for consent for optional follow-up visits every week for the next month, then monthly from enrollment. While participants were offered the option of a single visit, 99% participated in longitudinal sampling; study samples were obtained regularly, irrespective of the presence or absence of symptoms. Supplementary Table 1 provides a detailed description of the number of visits per participant, with a median of 18 visits (interquartile range [IQR], 12–21) between 1 October 2020 and 23 April 2022.

Swab samples were analyzed at national Lighthouse Laboratories at Milton Keynes and Glasgow, using identical methods. PCR for 3 SARS-CoV-2 genes (N protein, S protein and open reading frame (ORF)1ab) was performed using the Thermo Fisher TaqPath RT-PCR coronavirus disease 2019 (COVID-19) kit, and analyzed using UgenTec FastFinder 3.300.5, with an assay-specific algorithm and decision mechanism that allows conversion of amplification assay raw data into test results with minimal manual intervention. Samples are called positive if at least the N-gene and/or ORF1ab are detected. Although S-gene cycle threshold values are determined, S-gene detection alone is not considered sufficient to call a sample positive, according to the assay manufacturer [8].

The presence of 12 specific symptoms in the previous 7 days was elicited at each visit from the start of the survey (cough, fever, myalgia, fatigue/weakness, sore throat, shortness of breath, headache, nausea, abdominal pain, diarrhea, loss of taste, loss of smell), as was whether participants thought they had (unspecified) symptoms compatible with COVID-19. Positive response to any of these questions defined “symptomatic” cases. Four additional symptoms (runny nose, trouble sleeping, loss of appetite, wheezing) were added from 29 September 2021; because these were not elicited throughout the survey, they were considered separately and not used to define symptomatic cases.

**Figure 1.**
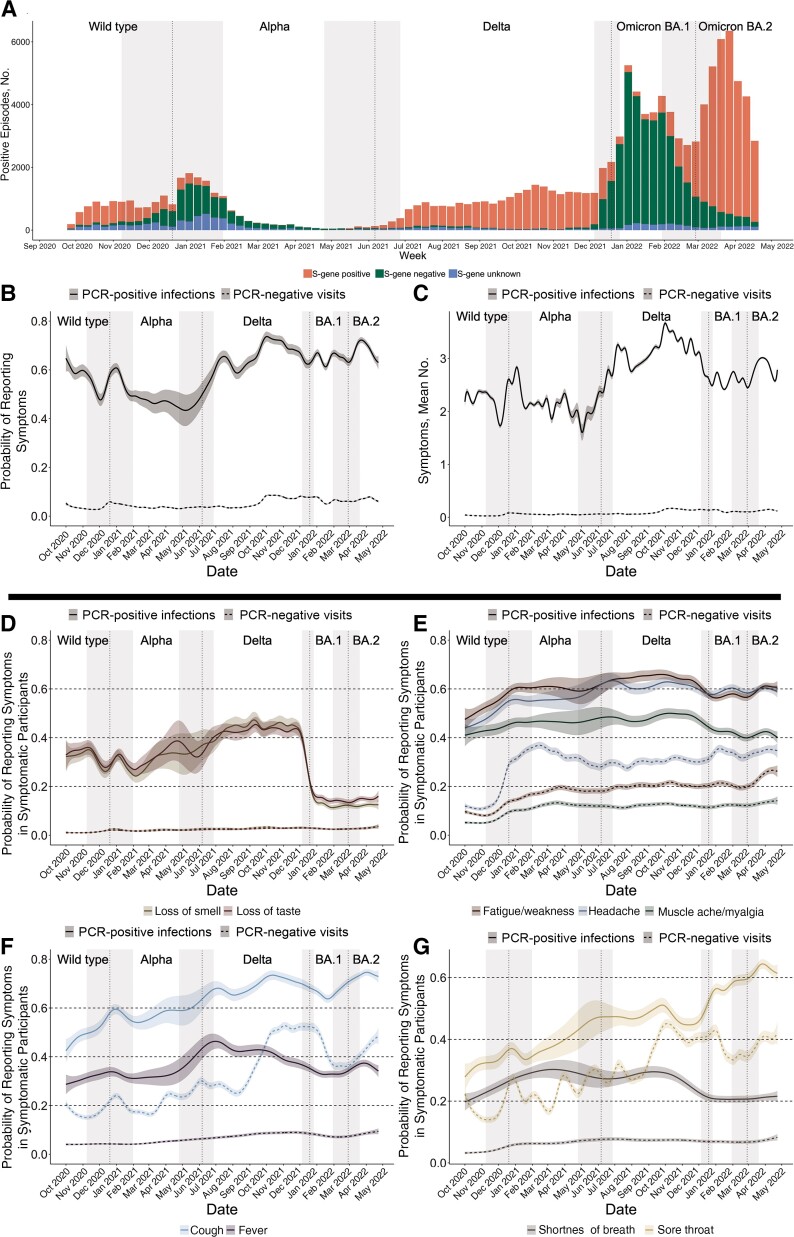
Variants (*A*) and symptoms (*B*–*G*) in participants testing positive or negative for severe acute respiratory syndrome coronavirus 2 (SARS-CoV-2) over time in the United Kingdom. *A*, Numbers of polymerase chain reaction (PCR)–positive infection episodes that were S-gene negative (Alpha compatible, 20 December 2020 to 5 June 2021; Omicron BA.1 compatible, 19 December 2021 to 26 February 2022) or S-gene positive (Delta compatible, 6 June to 18 December 2021; Omicron BA.2 compatible, 27 February 2022 onward). Vertical lines indicate when new variants became dominant based on gene positivity patterns (>50% of PCR-positive infection episodes, excluding those that were S-gene unknown): wild type before 20 December 2020, then Alpha before 5 June 2021, Delta before 19 December 2021, and Omicron BA.1 before 27 February 2022; Omicron BA.2 became the dominant variant afterward; while grey bands indicate periods between the first time when new variants represented >10% and >90% of PCR-positive infection episodes based on gene positivity patterns, excluding those that were S-gene unknown. *B, C*, Probability of reporting symptoms and the number of symptoms (of the 12 elicited throughout the study period) among all PCR-positive infection episodes and all PCR-negative comparator visits. *D–G*, Probability of specific symptoms in symptomatic PCR-positive infection episodes and symptomatic PCR-negative comparator study visits, after adjustment for age, sex, and ethnicity (presented at the reference categories of age 45 years, male sex, and white race).

We grouped repeated PCR-positive test results into infection “episodes” [11] and included the first positive study test in each episode in analysis (details in the Supplementary Methods). Each positive episode was characterized as wild-type, Delta, or Omicron BA.2 compatible if the S-gene was ever detected (by definition, with N-gene, ORF1ab, or both), or as Alpha or Omicron BA.1 compatible if positive at least once for both ORF1ab and N-gene (and never for the S-gene), and otherwise categorized as “other” (N-gene only/ORF1ab only), depending on calendar period (Figure 1*A*). Symptom presence was defined as reported symptoms at any visit within 35 days after the first PCR-positive result in each infection episode (ie, spanning from 7 days before to 35 days after the first PCR-positive result, given the question time frame), to allow for the random sampling leading to presymptomatic identification of some individuals, who reported symptoms only subsequently.

As a comparator, we initially considered all visits with negative PCR test results, and then, after a previous analysis to August 2021 [9], excluded visits for which symptoms could plausibly be related to ongoing effects of COVID-19 or long COVID, for which there was a high pretest probability of a new COVID-19 infection that had not been detected in the study, or for which symptoms were likely driven by recent vaccination (details in the Supplementary Methods).

Generalized additive models (binomial distribution with complementary log-log link) were fitted to estimate the percentage of PCR-positive infection episodes and PCR-negative visits for which participants were symptomatic, and the percentage of each of these for each symptom separately. Models adjusted simultaneously for calendar time (smoothing spline), age (smoothing spline), sex, and ethnicity (white vs nonwhite). From 29 September 2021 onward, fitted models with an additional interaction between age and time were used to present differences in symptoms by age.

To explore differences between Delta, Omicron BA.1, and Omicron BA.2 infections by vaccination status and infection/reinfection, we restricted PCR-positive infection episodes to those occurring after 29 September 2021 and classified S-gene–negative infections occurring after 1 December 2021 as Omicron BA.1 compatible (34 576 infections; 20 345 [59%] symptomatic), and S-gene–positive infections from 29 September 2021 to 2 January 2022 as Delta compatible (14 318 infections; 9030 [63%] symptomatic) and from 30 January to 23 April 2022 as Omicron BA.2 compatible (34 796 infections; 2 591 [65%] symptomatic) (excluding S-gene­–positive infections from 3 to 29 January 2022 because both Delta and Omicron BA.2 infections occurred during this period and genetic sequences were not available for all PCR-positive results). Descriptive analyses are presented of differences in symptom presence or absence and specific symptoms by variant, vaccination status, and infection episode. Comparisons by vaccine status are restricted to participants ≥18 years old to reduce confounding arising from lower vaccination rates in those <18 years old.

All analyses were performed using R 3.6.1 software. Generalized additive models were fitted using mgcv 1.8–31; example code is provided in the Supplementary Methods. Figures were produced using ggplot2 3.1.0 and cowplot 1.1.0 software.

## RESULTS

Between October 2020 and April 2022, a total of 120 995 PCR-positive episodes occurred in 115 886 participants (median age, 44 years; IQR, 24–61 years), 70 683 (58%) with reported symptoms; 8898 of 120 995 (7%) were reinfections (Supplementary Figure 1), 4244 (48%) with reported symptoms. The comparator comprised 4 766 366 PCR-negative study visits (483 894 participants; median age, 55 years; IQR, 36–68 years), 203 422 (4%) with reported symptoms.

While Omicron BA.1 infections dominated (19 December 2021 to 26 February 2022, when >50% of PCR-positive results were S-gene negative), the percentage of PCR-positive infection episodes with reported symptoms was lower compared with much of the previous time period when the Delta variant dominated (6 June to 18 December 2021; Figure 1*B* and *C*). Reporting of any symptoms increased again after Omicron BA.2 became the dominant variant (27 February 2022 onward, when >50% of PCR-positive results were S-gene positive). For both Omicron BA.1 and BA.2 the mean number of symptoms reported in PCR-positive infection episodes was lower than with Delta, but it was higher with BA.2 than BA.1. Changes in the percentage reporting any symptoms at PCR-negative visits, and the mean number of symptoms reported at PCR-negative visits, were much smaller over these time periods, with very slight increases from October 2021 onward, likely owing in part to other seasonal infections.

For specific symptoms, among symptomatic PCR-positive infection episodes, there was a marked decline in reported loss of taste/smell for both Omicron variants, BA.1 and BA.2, from high levels during the period when Delta dominated, from 44% reporting loss of taste/45% reporting loss of smell on 17 October 2021 (approximately peak Delta; Figure 1*A*), to 16%/13% on 2 January 2022 (approximately peak BA.1), with only very small changes thereafter, to 15%/12% on 27 March 2022 (approximately peak BA.2). Although loss of taste/smell was also less common with Alpha than with Delta, it was even less common with Omicron BA.1/BA.2 than with Alpha (Figure 1*D*). Loss of taste/smell remained extremely uncommon in symptomatic PCR-negative visits throughout (Figure 1*D*).

There were concurrent smaller, but significant, declines in symptomatic PCR-positive infection episodes with reported cough, fever, fatigue/weakness, myalgia, shortness of breath, or headache during December 2021, as Omicron BA.1 dominated (Figures 1*E–G*). As Omicron BA.2 became dominant, cough increased again, as did fever and fatigue/weakness to a lesser extent, while shortness of breath, myalgia, and headache remained at similar levels to those observed with BA.1 (Figures 1*E–G*). The main changes in the percentages of symptomatic PCR-negative visits where these specific symptoms were reported included a substantial increase in cough in October 2021, which then decreased in January 2022 from 52% to 36%, before increasing again to 48% by 23 April 2022 (Figure 1*G*), and increased in headache over December 2021 (from 30% to 35%) and in fatigue/weakness over March 2022 (from 20% to 26%) (Figure 1*E*).

In contrast to these declines in other symptoms as Omicron BA.1 dominated, sore throat became more commonly reported with BA.1 and increased further with BA.2, from 46% to 56% in symptomatic PCR-positive infection episodes during December 2021, increasing to 64% by April 2022. Similarly to cough, sore throat became more commonly reported at PCR-negative visits during October 2021, if anything dropping slightly in January 2022, from 43% to 33%, before increasing again to 42% by 23 April 2022 (Figure 1*G*). These changes were smaller for symptomatic PCR-negative visits than for symptomatic PCR-positive infection episodes; that is, they were insufficient to explain Omicron-associated increases in sore throat.

Gastrointestinal symptoms were reported infrequently in symptomatic PCR-positive infection episodes regardless of variant and were reported at similar frequencies at PCR-negative visits (Supplementary Figure 2). Reporting of runny nose generally followed reporting of sore throat, whereas other symptoms generally declined with Omicron BA.1/BA.2 (Supplementary Figure 2).

**Figure 2.**
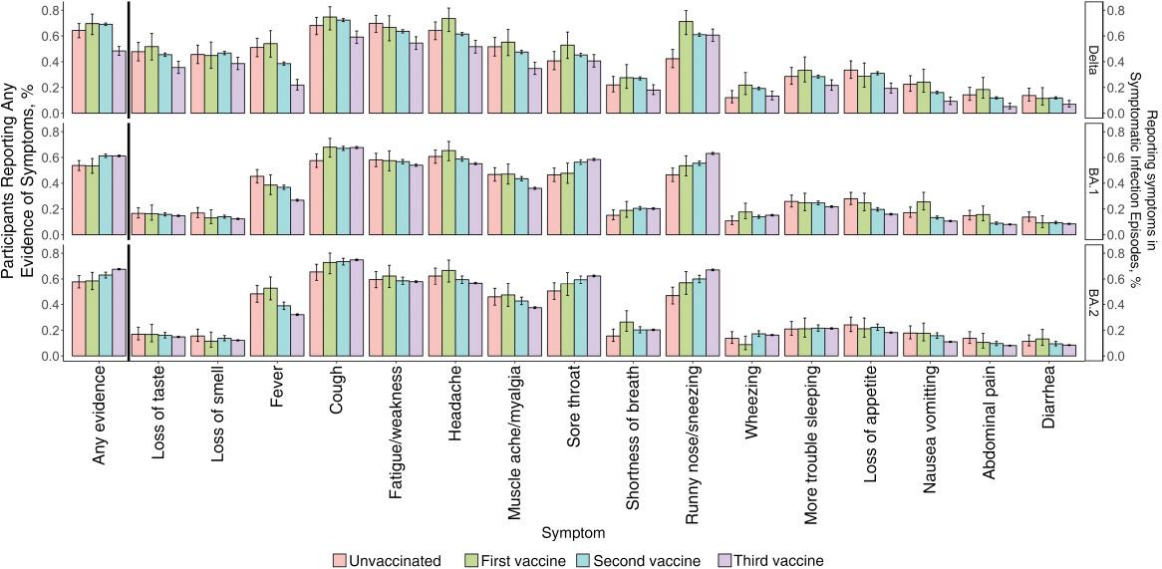
Percentage of polymerase chain reaction (PCR)–positive infection episodes reporting symptoms by variant and by vaccination status (restricting to those aged ≥18 years), showing reporting of any evidence of symptoms as well as specific symptoms in symptomatic PCR-positive infection episodes from 29 September 2021 onward (not adjusted for other factors; see Figure 4 for adjusted effect of age). Unvaccinated indicates before first vaccination at index positive test or never vaccinated; first vaccine, 21 days after first vaccination to 13 days after second; second vaccine, 14 days after second vaccination to 13 days after third; third vaccine, 14 days after third vaccination to 13 days after fourth (fourth vaccine data are not shown because these included <100 infections with evidence of symptoms; Supplementary Table 2). The unvaccinated and first vaccine groups represent only 3% of infections; these participants are potentially more likely to have been previously infected (because infection may have affected subsequent vaccine uptake), and previous infection is associated with fewer reported symptoms (Figure 3).

**Figure 3.**
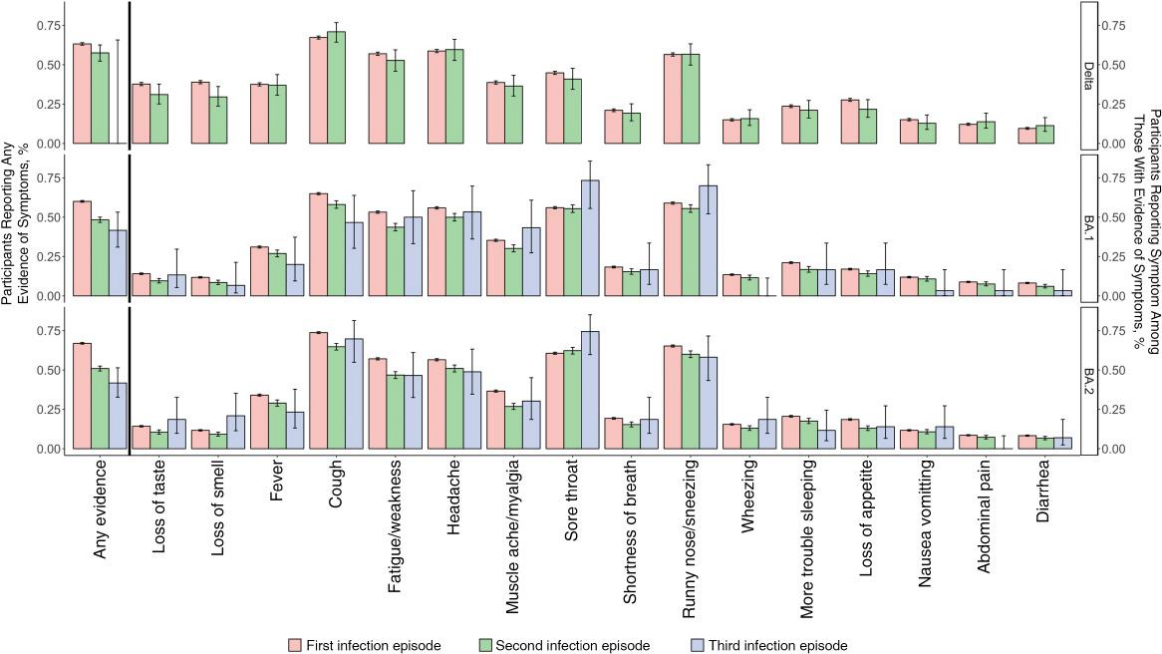
Percentage of polymerase chain reaction (PCR)–positive infection episodes reporting symptoms by variant and infection/reinfection, based on reporting of any evidence of symptoms, as well as specific symptoms in symptomatic PCR-positive infection episodes from 29 September 2021 onward (not adjusted for other factors; see Figure 4 for adjusted effect of age).

In participants aged ≥18 years, differences in symptoms between Delta and Omicron infections, including fewer cases with loss of taste/smell and more with sore throat, were broadly similar across all vaccination statuses (Figure 2, Supplementary Figure 3) (1304 [2%], 606 [1%], 14 706 [22%], and 49 981 [75%] of PCR-positive infection episodes occurred in those unvaccinated or vaccinated once, twice, or 3 times respectively; full split by variant and evidence of symptoms in Supplementary Table 2). Similarly, changes in symptoms by variant were also relatively unaffected by whether the PCR-positive infection episode was the first infection (91%) versus reinfection (9%) (Figure 3 and Supplementary Figure 4). However, overall, symptoms were less commonly reported in subsequent infections occurring from 29 September 2021 onward (50%), compared with first infections during this time period (63%), but specific symptoms were reported at broadly similar frequencies in participants who were symptomatic in PCR-positive first and subsequent infections with Delta and Omicron BA.1 and BA.2 variants.

**Figure 4.**
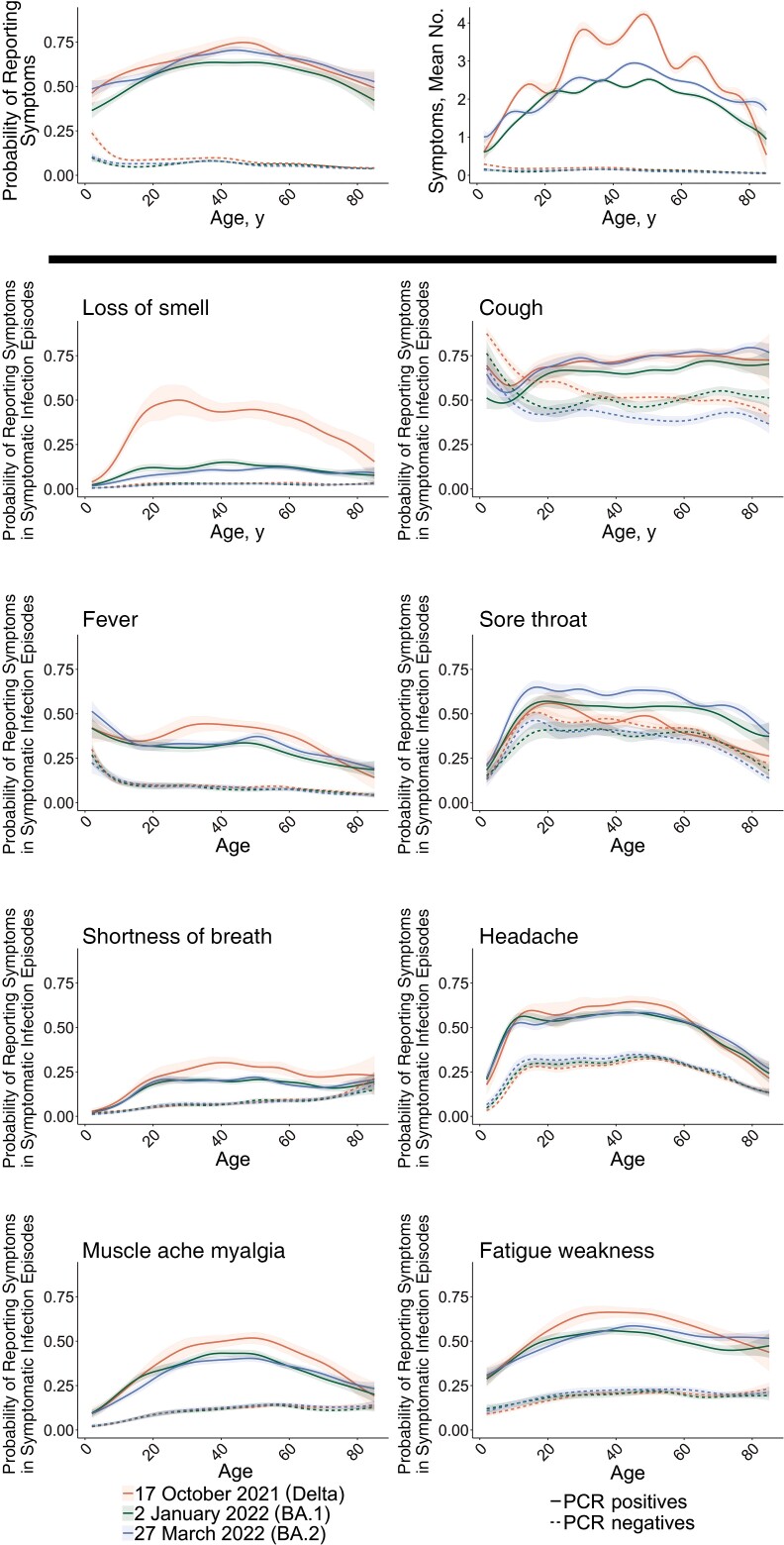
By age, estimated percentage of polymerase chain reaction (PCR)–positive infection episodes and comparator PCR-negative study visits reporting symptoms and mean number of symptoms at the peaks of Delta, Omicron BA.1, and Omicron BA.2 waves. Model estimates are shown for reporting of any evidence of symptoms as well as specific symptoms in symptomatic PCR-positive infection episodes and comparator PCR-negative study visits on 17 October 2021 (Delta), 2 January 2022 (when Omicron BA.1-compatible infections represented the highest proportion of PCR-positive infections), and 27 March 2022 (when Omicron BA.2 was the dominant variant). Panels in the first row show the probability of reporting symptoms and the number of symptoms (of the 12 elicited throughout the study period) in all PCR-positive infection episodes and all PCR-negative comparator visits from 29 September 2021 onward, estimated at 3 reference categories, 17 October 2021, 2 January 2022, and 27 March 2022. The remaining panels show the probability of reporting specific symptoms in symptomatic PCR-positive infection episodes and in symptomatic PCR-negative comparator study visits at these reference categories. All are adjusted for calendar date, age (allowing for effect modification by calendar date by including an interaction between calendar date and age), sex (reference category: male), and ethnicity (reference category: white). See Supplementary Figure 3 for other symptoms.

There were differences in reported symptoms with these different variants by age when comparing reported symptoms at the peaks of the Delta, BA.1 and BA.2 waves (Figure 4, Supplementary Figure 5). Adults aged 18–65 years were more likely to report the presence of any symptoms than children or adults >65 years old. There was generally no evidence of difference in reporting the presence of any symptoms between Delta and BA.2, but there was a lower probability of reporting any symptoms with BA.1 across most ages. However, the mean number of symptoms reported with both BA.1 and BA.2 was generally lower across the ages compared with Delta, except in the youngest and oldest participants, for whom there was no evidence of difference in the mean number of symptoms between BA.1 and Delta but a higher mean number of symptoms for BA.2 than for Delta. Symptoms were less likely to be reported in PCR-positive infection episodes in children than in younger adults, even more so with Omicron BA.1 than with Delta and BA.2 (Supplementary Figure 6), whereas symptoms were most likely to be reported at PCR-negative visits in children, in particular cough and fever.

Loss of taste or smell was most commonly reported with Delta infections in adults aged 18–70 years but was reported at lower levels in older adults and rarely in younger children; with Omicron BA.1/BA.2 infections, it was seen only at low levels, regardless of age. Variations in the percentage of symptomatic participants reporting most other specific symptoms across ages were broadly similar before versus after dominance of Omicron BA.1, but slightly higher percentages of participants >70 years of age with symptomatic PCR-positive infection episodes reported fever, headache, fatigue/weakness, or muscle ache/myalgia after Omicron BA.1/BA.2 dominated (Figure 4). Most specific symptoms were reported less frequently with infections in young children compared with adolescents/young adults, regardless of the dominating variant, with the exception of fever, which was reported significantly more with Omicron BA.1 and BA.2 infections in young children than in adolescents/young adults, particularly for BA.2 (Supplementary Figure 6).

The net result of changes in the symptom profile, overall and by age, was that fever and cough became most strongly associated with PCR positivity in those reporting symptoms after Omicron BA.2 became dominant, adjusting for age, sex, and ethnicity (see Supplementary Methods and Supplementary Figure 7). Although far less strongly associated than during the period when Delta was the main variant, loss of taste was still the fourth most strongly associated symptom after Omicron BA.2 dominated, with fatigue/weakness also strongly associated. These same 4 symptoms were also most strongly associated with PCR positivity when Omicron BA.1 dominated. Sore throat was positively associated with PCR positivity during the BA.2-dominant period, and to a slightly lesser during the BA.1-dominant period; in contrast, sore throat was less likely to occur in symptomatic PCR-positive infection episodes than in symptomatic PCR-negative visits during the Delta period.

## DISCUSSION

In this study of predominantly mild community-based infection, Omicron BA.1 and BA.2, compared with Delta, were associated with less loss of taste, loss of smell, shortness of breath, myalgia, fatigue/weakness, and headache but more sore throat. The overall probability of reporting any symptoms was similar for Delta and BA.2 but lower for BA.1 regardless of age, while the mean number of symptoms reported was generally lower for both BA.1 and BA.2 compared with Delta across ages, although higher overall for BA.2 than BA.1. However, this was driven by symptoms in adults; in the youngest and oldest participants, there was no evidence of difference between BA.2 and Delta in the percentage reporting any symptoms, and a higher mean number of symptoms was reported with BA.2 in the very youngest and oldest participants, compared to both BA.1 and Delta.

In PCR/lateral flow antigen–positive cases, the ZOE study, which relies on volunteers reporting symptoms daily using an app, found a lower median number of symptoms reported in infections from 28 November 2021 to 17 January 2022 (predominantly Omicron BA.1) than from 1 June to 27 November 2021 (predominantly Delta), with matching by age, sex, and ethnicity in volunteers who had had a second or third vaccine [12] and with less loss of smell and more sore throat reported with Omicron BA.1, as in our study. The major strength of our study is that regular PCR testing was undertaken in all participants at all visits irrespective of symptoms.

This provides a representative sample of PCR-negative visits without SARS-CoV-2 infection for comparison with symptom rates for PCR-positive infection episodes. This is important because some symptoms reported in PCR-positive infections could be due to coinfections with other circulating respiratory viruses. Therefore, although our study does not specifically test for other viruses, we can estimate whether changes seen with Omicron BA.1 and BA.2 differ from underlying trends in the general population (Figures 1*D–*1*G*), supporting the hypothesis that much of the increase in sore throat is attributable to Omicron rather than other infections. We are also able to demonstrate large shifts in symptoms reported at PCR-negative visits over time, with concurrent increases in cough and sore throat in October 2021 likely reflecting other respiratory viruses. We also note that the probability of reporting any symptoms, as well as specific symptoms, varied considerably during the periods when specific variants dominated, potentially reflecting how the survey captures more infections earlier on when positivity is rising, and more later on as positivity is decreasing [13]. We compared rates at the peak of each dominating variant to capture similar phases of the epidemic, as well as considering how these changed over time.

Intriguingly, we found that the differences between variants in the probability of reporting specific symptoms in symptomatic PCR-positive infection episodes persisted regardless of vaccination status or whether the infection was the first or a subsequent infection, while the probability of reporting symptoms was smaller for reinfections than for first infections. A limitation is that this analysis is of unadjusted percentages, and therefore the lack of observed differences by vaccination status within a variant could be at least partly due to confounding with age, as well as other factors, such as previous infection, which could lead to choosing not to be vaccinated or to get only a single vaccine (only 3% of the infections included in this analysis). However, most symptoms were reported similarly in adults aged 18 to about 60–70 years (Figure 4).

Other limitations of the current study include the fact that we cannot have certainty in determining reinfections given the data available; however, estimated reinfections were infrequent (7%), even once Omicron dominated (11%), and symptom profiles were broadly similar in first and subsequent infections from 29 September 2021. Another limitation is that the study does not collect data on healthcare provider visits, hospitalizations, or death, to allow analysis of the severity of Omicron infections beyond reported symptoms. The ZOE study found lower self-reported hospitalization rates with infections occurring during the Omicron BA.1-dominant versus the Delta-dominant period and shorter duration of symptoms [12], and several other studies have documented lower hospitalization rates with Omicron BA.1 [14–17].

Increases in sore throat (also commonly reported at symptomatic PCR-negative visits) and the marked reduction in the previously highest-specificity symptoms—namely, loss of taste/smell—present challenges for testing algorithms. Previously during periods when wild-type virus or Alpha and Delta variants dominated, fever, cough, or loss of taste/smell have been shown to offer a good balance between sensitivity and specificity for detecting SARS-CoV-2 infections [9]. In the United Kingdom, for much of the pandemic to date, any of these 4 symptoms formed a basis for the general public accessing PCR testing. However, changes in symptoms with Omicron mean that symptom-based screening for testing is now much more difficult, and these changes have resulted in much broader criteria for symptoms suggestive of COVID-19 being proposed [18], albeit with likely decreased specificity. In conclusion, changes in SARS-CoV-2 infection symptoms mean that Omicron is harder to detect with symptom-based testing algorithms, with implications for institutional and national testing policies.

## Supplementary Data

Supplementary materials are available at *Clinical Infectious Diseases* online. Consisting of data provided by the authors to benefit the reader, the posted materials are not copyedited and are the sole responsibility of the authors, so questions or comments should be addressed to the corresponding author.

## Supplementary Material

ciac613_Supplementary_Data

## Notes

***Author contributions.*** K. D. V., K. B. P., T. E. A. P., T. H., D. C., P. C. M., N. S., D. W. E., and A. S. W. designed the specific analysis. K. D. V. conducted the statistical analysis of the survey data. K. D. V., D. E., and A. S. W. drafted the manuscript. All authors contributed to interpretation of the study results, revised and approved the manuscript for intellectual content, and had full access to all data analysis outputs (reports and tables) and take responsibility for their integrity and accuracy. K. D.V. is the guarantor and accepts full responsibility for the work and conduct of the study, had access to the data, controlled the decision to publish, and attests that all listed authors meet authorship criteria and that no others meeting the criteria have been omitted.

***Acknowledgments.*** We are grateful for the support of all COVID-19 Infection Survey participants and the members of the COVID-19 Infection Survey team, listed here. Office for National Statistics: I. D., E. R., Ruth Studley, Tina Thomas, and D. Cook; Survey Analysis and Operations teams, especially Daniel Ayoubkhani, Russell Black, Antonio Felton, Megan Crees, Joel Jones, Lina Lloyd, and Esther Sutherland; University of Oxford, Nuffield Department of Medicine: A. S. W., D. Crook, P. C. M., T. E. A. P., E. P., N. S., K. D. V., Jia Wei, Alison Howarth, George Doherty, James Kavanagh, Kevin K. Chau, Stephanie B Hatch, Daniel Ebner, Lucas Martins Ferreira, Thomas Christott, Brian D. Marsden, Wanwisa Dejnirattisai, Juthathip Mongkolsapaya, Sarah Cameron, Phoebe Tamblin-Hopper, Magda Wolna, Rachael Brown, Sarah Hoosdally, Richard Cornall, Yvonne Jones, David I Stuart, and Gavin Screaton; University of Oxford, Nuffield Department of Population Health: K. B. P. University of Oxford, Big Data Institute: D. W. E., Katrina Lythgoe, David Bonsall, Tanya Golubchik, and Helen Fryer; University of Oxford, Radcliffe Department of Medicine: John Bell; Oxford University Hospitals NHS Foundation Trust: Stuart Cox, Kevin Paddon, and Tim James; University of Manchester: T. H.; UK Health Security Agency: John Newton, Julie Robotham, and Paul Birrell; IQVIA: Helena Jordan, Tim Sheppard, Graham Athey, Dan Moody, Leigh Curry, and Pamela Brereton; National Biocentre: Ian Jarvis, Anna Godsmark, George Morris, Bobby Mallick, and Phil Eeles; Glasgow Lighthouse Laboratory: Jodie Hay and Harper VanSteenhouse; Department of Health and Social Care: Jessica Lee; Welsh Government: Sean White, Tim Evans, and Lisa Bloemberg; Scottish Government: Katie Allison, Anouska Pandya, and Sophie Davis; Public Health Scotland: David I. Conway, Margaret MacLeod, and Chris Cunningham.

***Disclaimer.*** The views expressed are those of the authors and not necessarily those of the National Institute for Health Research, UK Health Security Agency or the Department of Health and Social Care. The funders/sponsors did not have any role in the design and conduct of the study; collection, management, analysis, and interpretation of the data; preparation, review, or approval of the manuscript; or decision to submit the manuscript for publication. For the purpose of Open Access, the authors have applied a CC BY public copyright license to any author accepted manuscript version arising from this submission.

The lead authors (K. D. V., D. W. E., A. S. W.) affirm that the manuscript is an honest, accurate, and transparent account of the study design being reported, that no important aspects of the study have been omitted, and that any discrepancies from the study as originally planned (and, if relevant, registered) have been explained. Results of individual tests were communicated to the participants. Overall study results were disseminated through the preprint of the study.

***Financial support.*** This work was supported by the Department of Health and Social Care with in-kind support from the Welsh Government; the Department of Health on behalf of the Northern Ireland Government; the Scottish Government; the National Institute for Health Research (NIHR) Health Protection Research Unit in Healthcare Associated Infections and Antimicrobial Resistance, University of Oxford, in partnership with UK Health Security Agency (grant NIHR200915 to K. D. V., K. B. P., T. E. A. P., N. S., A. S. W., and D. W. E.); the NIHR Oxford Biomedical Research Centre (support to T. E. A. P. and A. S. W.; senior fellowship awards to N. S. and D. W. E.); the Huo Family Foundation (K. B. P.); core support from the Medical Research Council UK to the MRC Clinical Trials Unit (MC_UU_12023/22 to A. S. W.); the NIHR (support to A. S. W. as a senior investigator); Wellcome (intermediate fellowship; grant 110110/Z/15/Z to P. C. M.); the Robertson Foundation (Robertson Fellowship to D. W. E.); and the Oxford Martin School (fellowship to N. S.)

***Data sharing.*** Data are still being collected for the COVID-19 Infection Survey. Deidentified study data are available for access by accredited researchers in the Office for National Statistics Secure Research Service (SRS) for accredited research purposes under part 5, chapter 5, of the Digital Economy Act 2017. For further information about accreditation, contact Research.Support@ons.gov.uk or visit the SRS Web site https://www.ons.gov.uk/aboutus/whatwedo/statistics/requestingstatistics/secureresearchservice.
